# Effect of connective tissue growth factor (CCN2/CTGF) on proliferation and differentiation of mouse periodontal ligament-derived cells

**DOI:** 10.1186/1478-811X-3-11

**Published:** 2005-10-05

**Authors:** Masahiro Asano, Satoshi Kubota, Tohru Nakanishi, Takashi Nishida, Tomoichiro Yamaai, Gen Yosimichi, Kazumi Ohyama, Tomosada Sugimoto, Yoji Murayama, Masaharu Takigawa

**Affiliations:** 1 Department of Biochemistry and Molecular Dentistry, Okayama University Graduate School of Medicine, Dentistry and Pharmaceutical Sciences, Okayama, Japan; 2 Department of Periodontal Science, Okayama University Graduate School of Medicine, Dentistry and Pharmaceutical Sciences, Okayama, Japan; 3 Department of Oral Functional Anatomy, Okayama University Graduate School of Medicine, Dentistry and Pharmaceutical Sciences, Okayama, Japan

## Abstract

**Background:**

CCN2/CTGF is known to be involved in tooth germ development and periodontal tissue remodeling, as well as in mesenchymal tissue development and regeneration. In this present study, we investigated the roles of CCN2/CTGF in the proliferation and differentiation of periodontal ligament cells (murine periodontal ligament-derived cell line: MPL) *in vitro*.

**Results:**

In cell cultures of MPL, the mRNA expression of the CCN2/CTGF gene was stronger in sparse cultures than in confluent ones and was significantly enhanced by TGF-β. The addition of recombinant CCN2/CTGF (rCCN2) to MPL cultures stimulated DNA synthesis and cell growth in a dose-dependent manner. Moreover, rCCN2 addition also enhanced the mRNA expression of alkaline phosphatase (ALPase), type I collagen, and periostin, the latter of which is considered to be a specific marker of the periosteum and periodontium; whereas it showed little effect on the mRNA expression of typical osteoblastic markers, e.g., osteopontin and osteocalcin. Finally, rCCN2/CTGF also stimulated ALPase activity and collagen synthesis.

**Conclusion:**

These results taken together suggest important roles of CCN2/CTGF in the development and regeneration of periodontal tissue including the periodontal ligament.

## Background

Connective tissue growth factor (CCN2/CTGF) is a cysteine-rich protein with a molecular weight of 36–38 kDa [1-3] and belongs to the CCN family, which stands for CTGF, CEF10/Cyr61, and Nov [4]. This family now consists of 6 distinct members [1-3], including additional members that had been named WISP1/ELM1, WISP2/COP-1, and WISP3. These proteins retain structural similarity to one another, characterized by an N-terminal secretory signal, followed by 4 modules, with the exception of COP-1, which lacks the C-terminal module [1-4]. As such, CCN proteins share 30–50% over all amino acid sequence homology and 40–60% nucleotide sequence homology with each other [1-4]. The members of this family exhibit functional diversity, being involved in such cellular processes as adhesion, migration, proliferation and differentiation, and in biological processes such as angiogenesis and chondrogenesis [1-3].

CCN2/CTGF was discovered as a chemotactic and mitogenic factor for fibroblast-like cells *in vitro *and so was named accordingly [5]. It was also found in skin fibroblasts *in vivo *as a repairing growth factor [6]. Of note, transforming growth factor (TGF)-β was shown to act in tissue during normal wound repair [6,7] and fibrotic disorders of the skin [8,9], and in internal organs and tissues [10-12]. CCN2/CTGF was also shown to be closely linked to the expression of TGF-β in these situations [6].

CCN2/CTGF increased the expression of extracellular matrix molecules such as type I collagen, fibronectin, and integrin [13], and was overexpressed in fibroblasts in the dermis of patients with scleroderma [14] or other fibrotic disorders [15-18]. These findings suggest that CCN2/CTGF plays an important role in cell proliferation and matrix synthesis in connective tissue.

Critical roles of CCN2/CTGF in other mesenchymal tissues have been uncovered as well. Our previous studies revealed that *ccn2/ctgf *was highly expressed in hypertrophic chondrocytes in growth cartilage and human chondrocytic HCS-2/8 cells [19]. CCN2/CTGF actually stimulated the proliferation and differentiation of chondrocytes *in vitro *[20] and induced articular cartilage regeneration *in vivo *[21]. Importantly, CCN2/CTGF also promotes the proliferation, migration, and tube formation of vascular endothelial cells *in vitro *and angiogenesis *in vivo *[22,23]. Such biological effects of CCN2/CTGF were further confirmed by the abnormal phenotypic change in the growth plate of CCN2/CTGF knockout mice. Moreover, roles of CCN2/CTGF in the proliferation and differentiation of osteoblasts have been also indicated [24]. In a recent study, regulated expression of CCN2/CTGF in developing tooth germs was reported [25,26]. During odontogenesis, CTGF/CCN2 gene expression was clearly observed not only in the dental mesenchyme but also in the dental epithelium up to the stage of enamel secretion [25,26]. Therefore, CCN2/CTGF is now regarded as a common regulator of the development of the 2 hard tissues in vertebrates – tooth and bone.

The periodontal ligament is a fibrous tissue located between these 2 mineralized hard tissues, and plays an important role in anchoring tooth and alveolar bone and in maintaining their structural integrity; and it is also involved in the periodontal regeneration process. The ligament consists of a number of different cell types, such as fibroblasts, osteoblasts, osteoclasts, cementoblasts, and their precursors, and so on. In particular, fibroblasts in the periodontal ligament are considered to be multi-potential cells, or heterogeneous cell populations, which have the capacity to differentiate into other types of cells. In this respect, the periodontal ligament fibroblasts supposedly play a key role in the formation, maintenance, and regeneration of this connective tissue.

Therefore, in the present study, we focused on these periodontal ligament fibroblasts by evaluating the effects of CCN2/CTGF on the *in vitro *biological phenotypes of a mouse periodontal ligament cell line (MPL).

## Results

### Expression of *ccn2/ctgf *mRNA in different growth phases in MPL cell cultures

**Figure 1:**
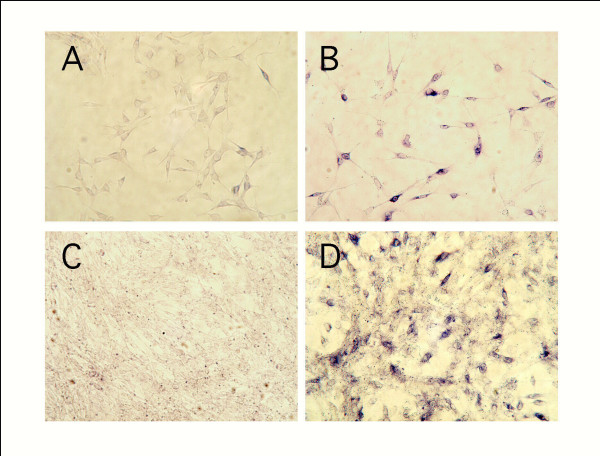
**Expression of CCN2/CTGF mRNA in sparse and confluent MPL cultures (in situ hybridization) with or without TGF-β stimulation. **MPL cultures in the sparsity phase (A and B) and confluence phase (C and D) were treated with TGF-β (B and D) or PBS (control: A and C). The cells were then cultured for 12 hrs, fixed, and hybridized with an antisense riboprobe for *ccn2/ctgf *mRNA. CCN2/CTGF mRNA was distinctly expressed in the cells in the sparse (A), but not in those in the confluent (C) state. However, the addition of TGF-β enhanced the level of CCN2/CTGF mRNA strongly, not only in the sparse cultures (B), but also in the confluent ones (D).

Initially, the expression of *ccn2/ctgf *mRNA in different phases of MPL growth was analyzed by *in situ *hybridization analysis. Positive signals were significantly observed in sparse cultures, but not in confluent ones. However, stronger mRNA expression was induced by the addition of TGF-β, even in cultures in the confluent state (Fig. 1).

**Figure 2:**
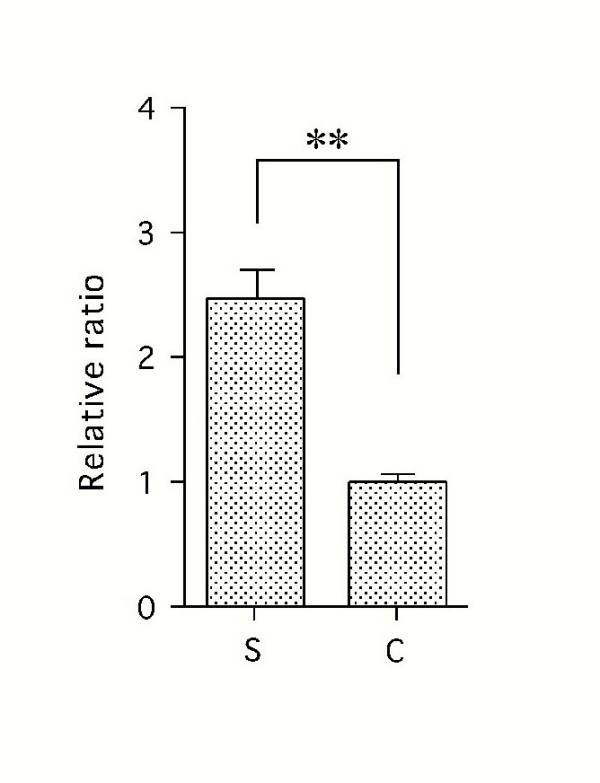
**RT-PCR analysis of the expression of CCN2/CTGF mRNA in sparse (S) and confluent (C) MPL cultures. **Total RNA was purified from MPL cultures under sparse and confluent conditions, and 1 μg in each sample was reverse transcribed and used for PCR. The expression of CCN2/CTGF mRNA was stronger under the sparse than the confluent condition as evaluated in the exponential phase of the amplification (25 cycles). Levels of GAPDH mRNA, used as an internal control, were almost the same among these samples.

Since strong expression of *ccn2/ctgf *mRNA was detected in sparse cultures of MPL by *in situ *hybridization analysis, we subsequently evaluated the expression level in different growth phases by semi-quantitative RT-PCR. The steady-state mRNA level in the sparse cultures of MPL cells was approximately 3 times higher than that in the confluent ones (Fig. 2). These results suggest the involvement of CCN2/CTGF and TGF-β in MPL cell proliferation on demand during the regeneration of periodontal ligament.

### Effects of rCCN2/CTGF on DNA synthesis and cell proliferation

**Figure 3:**
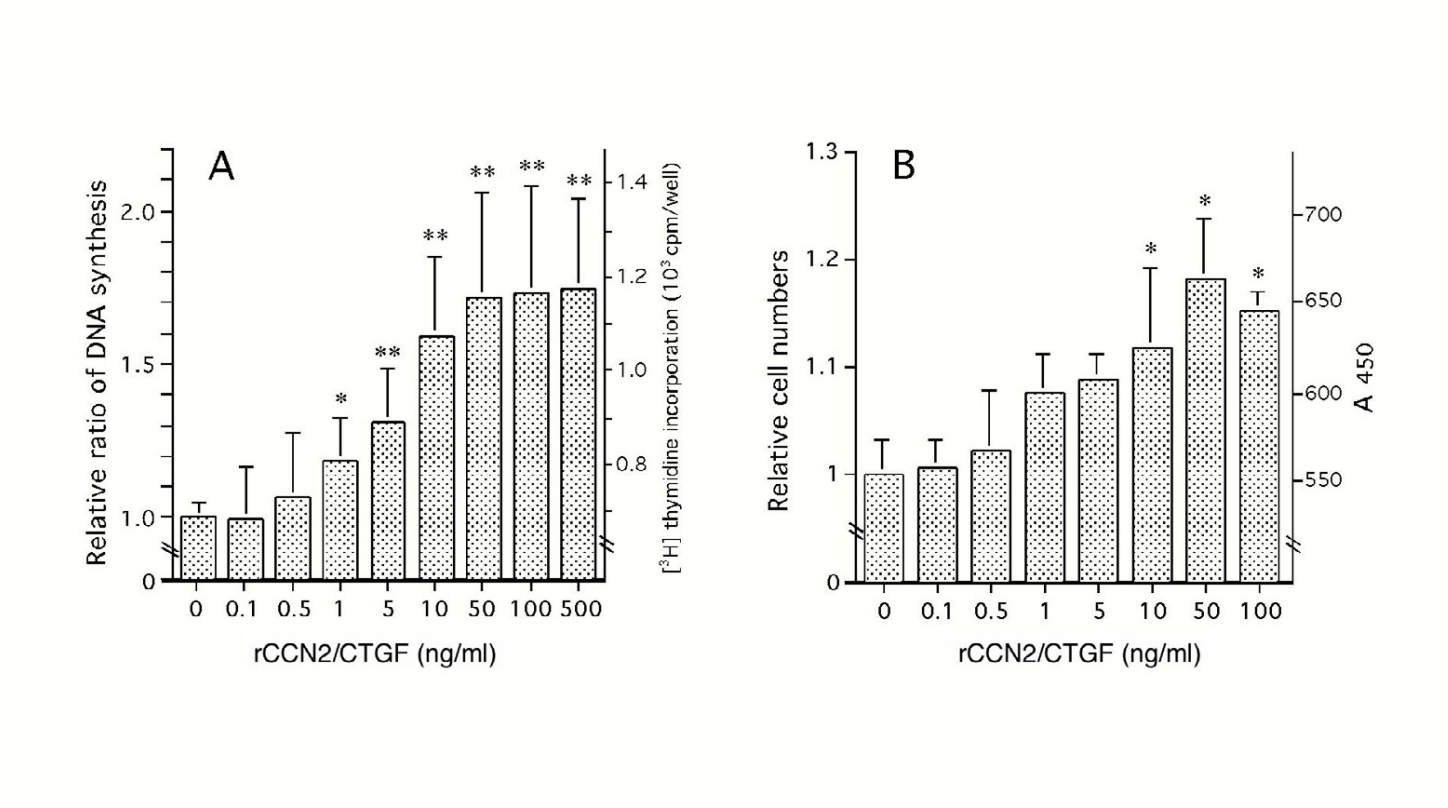
**(A) Effect of rCCN2/CTGF on DNA synthesis in MPL cells. **MPL cells were inoculated at a density of 5 × 10^3^/well in 96-multi-well plates and cultured in α-MEM with 10% FBS for 2 days. Then, the culture medium was changed to α-MEM with 0.5% FBS, and the cells were incubated with various concentrations of rCCN2/CTGF for 24 hrs and labeled with [^3^H] thymidine for the last 4 hrs. The incorporated radioactivity was determined by a liquid scintillation counter. Values represent the averages ± SD. Data were computed with the results of 2 independent series of experiments with multiple sample numbers. Asterisks denote statistically significant difference from the vehicle-treated control. **(B) Effect of rCCN2/CTGF on the proliferation of MPL cells. **MPL cells were inoculated at a density of 2 × 10^3^/well in 96-multi-well plates and cultured in α-MEM with 10% FBS for 24 hrs. Then, the medium was changed to α-MEM with 1% FBS, and the cells were cultured with various concentrations of rCCN2/CTGF for 3 days. The cell number was computed by using Tetra-Color One as specified by the manufacturer. Points and bars represent the averages and SD, respectively. Asterisks indicate significant differences between vehicle-treated control and rCCN2/CTGF-treated samples at the significance level of **p < 0.01 or *p < 0.05.

Based on the findings above, we next evaluated the effects of exogenous rCCN2/CTGF on MPL cell growth. As shown in Fig. 3A, rCCN2/CTGF stimulated DNA synthesis in the cells in a dose-dependent manner, as evaluated by [^3^H] thymidine incorporation. Significant effects were observed at 1 ng/ml and higher concentrations of rCCN2/CTGF. Similarly, the addition of rCCN2/CTGF increased the cell number under standard cell culture conditions after 3 days of treatment (Fig. 3B). The maximum effect produced a 1.2-fold increase over the control.

### Effects of rCCN2/CTGF on expression of genes related to the differentiated phenotypes of periodontal ligament (PDL) cells

**Figure 4:**
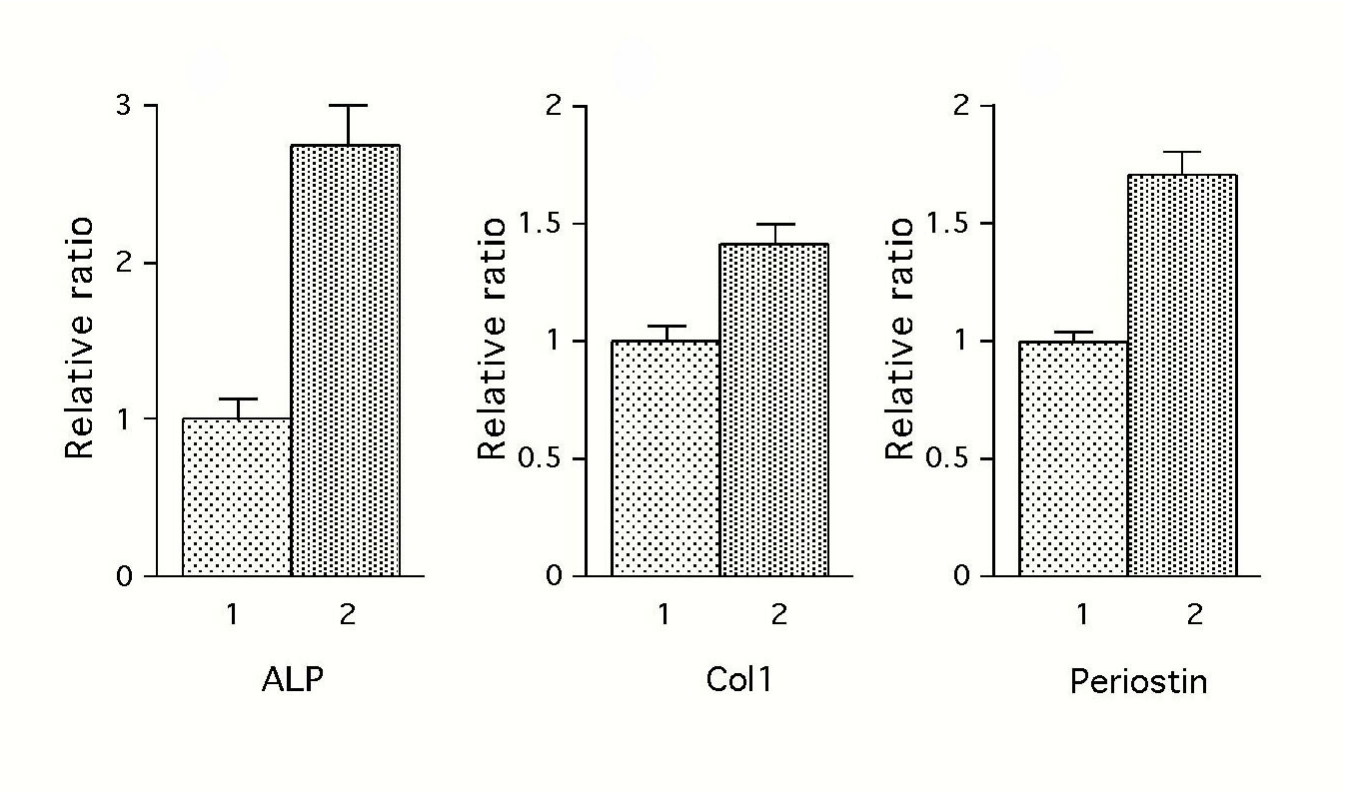
**Effects of rCCN2/CTGF on mRNA expression of osteoblast and PDL-related markers in MPL cells. **Confluent cultures of MPL cells were treated with rCCN2/CTGF (100 ng/ml, closed bars) or vehicle (dotted bars) for 24 hrs. Total RNA was purified, and 1 μg of each sample was reverse-transcribed and used for PCR. Alkaline phosphatase (ALPase), type I collagen, and periostin mRNAs were shown to be up-regulated clearly by the addition of rCCN2/CTGF under exponential conditions (25, 30, and 25 cycles, respectively) for amplification. The expression of GAPDH, used for an internal control, was almost the same among these samples.

In order to examine the effects of rCCN2/CTGF on gene expression of known mesenchymal differentiation markers, we conducted RT-PCR analysis. The mRNA expression of ALPase and type I collagen, which are considered to be phenotypic markers of periodontal ligament (PDL) cells as well as of osteoblasts, was up-regulated 24 hrs after the addition of 100 ng/ml of rCCN2/CTGF (ALPase: about 2.8-fold, type I collagen: about 1.5-fold; Fig. 4). Moreover, the expression of periostin, which is a marker of periosteal cells as well as of PDL cells, was also up-regulated approximately 2.5-fold 24 hrs after the addition of rCCN2/CTGF. On the other hand, the addition of rCCN2/CTGF had little effect on the mRNA expression of bone-specific phenotypic marker genes encoding osteopontin and osteocalcin (data not shown). The amount of GAPDH transcript, used as an internal control, was almost the same with or without rCCN2/CTGF.

### Effect of rCCN2/CTGF on ALPase Activity displayed by MPL cells

**Figure 5:**
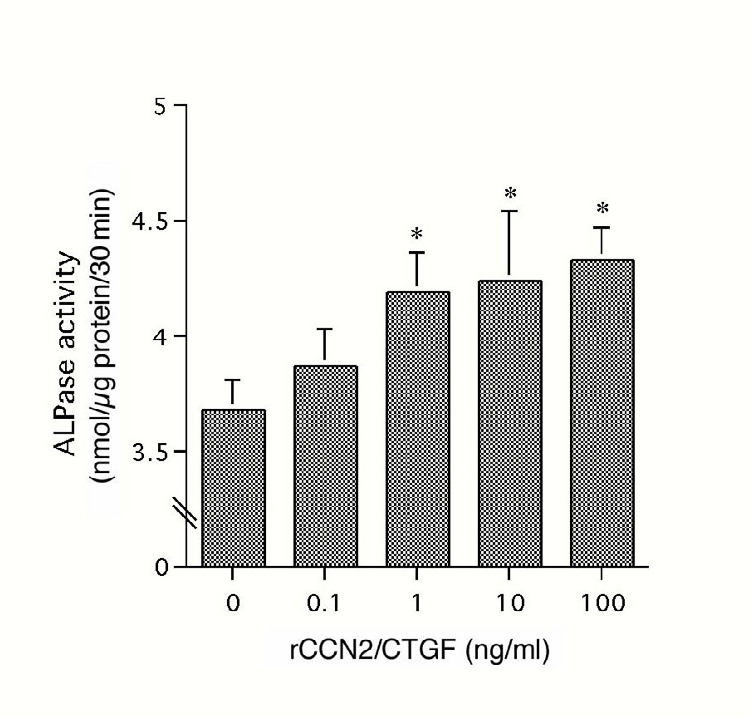
**Effects of rCCN2/CTGF on ALPase activity from MPL cells. **When MPL cells reached confluence, the culture medium was replaced with α-MEM containing 1% FBS, and the cells were then cultured with various concentrations of rCCN2/CTGF. After 48 hrs, the cell layers were collected, and ALPase activity was determined as described in "Materials and Methods." Values represent the averages ± SD of 3 separate experiments. Asterisks denote statistically significant differences from the vehicle-treated control at the significance level of *p < 0.01.

In addition to the examination of the mRNA level, we also investigated the effect of rCCN2/CTGF on ALPase activity displayed by MPL cells. As shown in Fig. 5, the enzyme activity increased in a dose-dependent manner 48 hrs after the addition of rCCN2/CTGF. A significant effect was observed at a dose as low as 1 ng/ml, reaching a plateau at 100 ng/ml.

### Effect of rCCN2/CTGF on collagen synthesis by MPL cells

**Figure 6:**
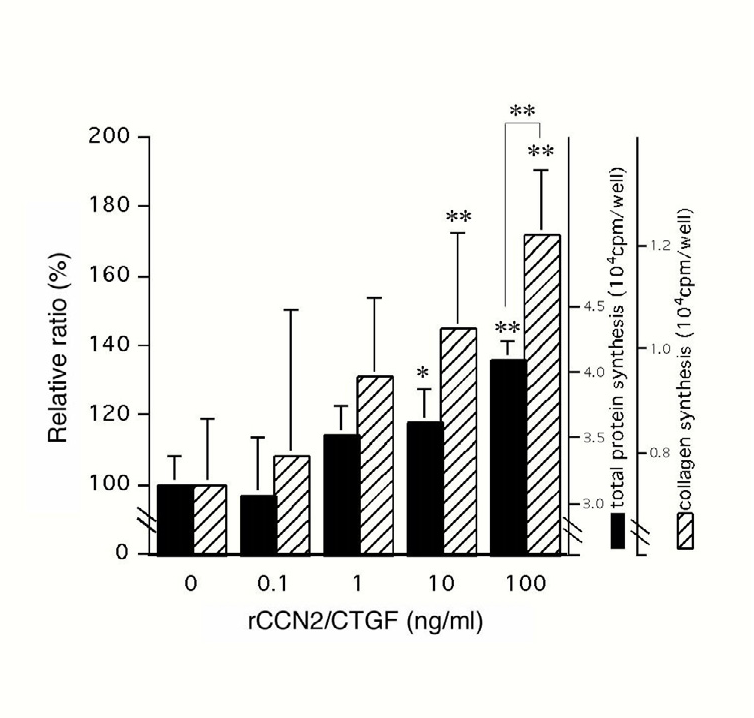
**Effect of rCCN2/CTGF on the synthesis of collagen and total protein in MPL cells. **Various concentrations of rCCN2/CTGF were added to confluent cultures of MPL cells, and the cells were cultured for 12 hrs. Then, the cells were labeled with [^3^H] proline for another 12 hrs, after which their cell layers were collected for analysis. The radioactivities of [^3^H] proline incorporated into total nascent proteins and collagenase-digestable portions were measured as described in "Materials and Methods." Slashed and closed boxes indicate the collagen and total protein synthesis, respectively. Values represent the mean average ± SD (n = 2). Asterisks denote statistically significant differences (**p < 0.01, *p < 0.05) from the vehicle-treated control, except the double asterisks specifying a significant difference between the stimulation levels of total protein and collagen synthesis (as indicated by the bracket). Data were computed with the results of 2 independent series of experiments with multiple sample numbers.

Since the predominant component of the extracellular matrix of the periodontal ligament is type I collagen, we further examined the effect of rCCN2/CTGF on collagen synthesis by MPL cells. As shown in Fig. 6, both total protein synthesis and collagen synthesis were increased by the addition of rCCN2/CTGF in a dose-dependent manner. However, the collagen-specific increase in [^3^H]-proline incorporation as estimated by collagenase digestion was significantly higher than that of the total increase. Therefore, CCN2/CTGF was indicated to promote the expression of PDL cell phenotypes, as well as overall protein synthesis, which might be the outcome of stimulated cell proliferation.

## Discussion

Active roles of CCN2/CTGF in tooth development and remodeling of periodontal tissues have been suggested by several reports that described the stage-specific expression of *ccn2/ctgf *with a particular spatial distribution in corresponding tissues [25,26]. In view of these reports, CCN2/CTGF is suggested to have some physiological function, playing a role in the organization and maintenance of periodontal tissues.

TGF-β, a major stimulator of the *ccn2/ctgf *gene, is expressed broadly in rat molar periodontal tissue in late embryos and after birth [36,37]. CCN2/CTGF expression was induced by TGF-β, but not by PDGF, EGF, or bFGF in cultured human foreskin fibroblasts [6]; and in a wound healing model, TGF-β and CCN2/CTGF mRNAs were coordinately expressed [6]. Therefore, as CCN2/CTGF has been shown to be closely linked to the expression of TGF-β, the CCN2/CTGF gene might be induced by TGF-β in periodontal tissue. Indeed, interaction between dental epithelium and mesenchyme was required for CCN2/CTGF induction, which could be substituted by exogenous TGF-β [25]. In our present study, TGF-β actually induced *ccn2/ctgf *expression in MPL cells and might be involved in the growth phase-dependent *ccn2/ctgf *expression. Therefore, interplay of TGF-β and CCN2/CTGF during tooth development is indicated as well.

The periodontal ligament lies between 2 hard tissues, alveolar bone and cementum, and consists of various kinds of cells. Owing to this specific situation, the major constituent cells – periodontal ligament fibroblasts – have unique characteristics unlike other fibroblasts. Periodontal ligament fibroblasts express calcification-related genes, and show ALPase activity. Although their functional significance is unclear, these phenotypes are thought to be critically important for fulfilling the specific missions of PDL cells.

As was shown in Fig. 1, positive signals for CCN2/CTGF mRNA in sparse MPL cultures were distinctly detected by *in situ *hybridization analysis, but were not clearly seen in confluent cells. We next evaluated the proliferative effect of rCCN2/CTGF on MPL cells. Regarding DNA synthesis, the incorporation of [^3^H] thymidine was increased in a dose-dependent manner by rCCN2/CTGF (Fig. 3A), and cell growth was stimulated in the same manner (Fig. 3B). These results suggest that CCN2/CTGF acts as an autocrine and/or paracrine cell proliferation-inducing factor. In addition, in our experiments, the stimulatory effect of rCCN2/CTGF on DNA synthesis was lost when MPL cells were cultured with α-MEM supplemented with 10% FBS, suggesting that more potent growth factors than rCCN2/CTGF are present in FBS.

In our RT-PCR investigation, the mRNA expression of ALPase and of type I collagen was up-regulated 24 hrs after the stimulation with rCCN2/CTGF (Fig. 4). In addition, rCCN2/CTGF stimulated the ALPase activity (Fig. 5). In the course of the calcification of hard tissues, including that of MPL cells in cell culture, the ALPase level is first up-regulated, the expression levels of osteopontin and osteocalcin are then up-regulated, and finally calcification occurs [38]. However, the gene expression of osteopontin and osteocalcin was not up regulated by CCN2/CTGF. Since we previously evaluated the cell biological effects of CCN2/CTGF on osteoblastic cells as well, the effects of CCN2/CTGF on the differentiation markers are comparatively summarized in Table 1. Such restricted expression of calcification-related genes may represent the specific phenotype of PDL cells, which discriminate them from osteoblasts. It should be also noted that the expression of *osf-2/cbfa1*, a master gene of osteoblastic differentiation, was not detectable in MPL cells. The observed effects of CCN2/CTGF to promote the proper differentiated phenotype of each type of the cells represent the characteristics of CCN2/CTGF as a modulator of extracellular signalling network.

**Table 1:** Expression profiles of the phenotypic markers in MPL *versus *osteoblastic cells

Cells	Basal *osf-2/cbfa1*	Induction by CCN2/CTGF			
		
		ALP	*opn*	*ocn*	*periostin*
MPL	-	+	-	-	+
MC3T3-E1*	+	+	+	+	N.D.

*Nishida et al. [24]

As for periodontal ligament fibroblasts, there is no clear differentiation marker that is specifically characteristic of this type of cell [39]. Thus, osteoblast-related markers have usually been used, although the ligament is an elastic tissue. However, in 1995, periostin cDNA was cloned from the MC3T3-E1 cell line by Takeshita et.al. [40]. They reported its stimulatory effect on cell-cell attachment and migration of MC3T3-E1 cells. Later, its limited expression was detected in both the periosteum and periodontium of teeth [41]. It was also reported that its strong expression in the periosteum was due to that in putative pre-osteoblasts and that this expression decreased as these pre-osteoblasts underwent maturation [40], indicating that periostin gene expression is a differentiation marker for immature, osteoblast progenitor cells. Gene expression of periostin is also detected in periodontium of teeth [41], which is also an elastic membranous tissue close to calcified-tissues, as is periostium, thus indicating that periostin gene expression is a marker of not only the periosteum but also of the periodontium. In other words, periostin is a marker of cells that are present close to calcified tissues and have potential to calcify but retain characteristics of an elastic, membranous tissue. Therefore, our finding that CCN2/CTGF potently stimulated periostin gene expression suggests that CCN2/CTGF stimulates the differentiation of these cells that constitute elastic, membranous tissues in between or close to calcified-tissues. Periostin is thought to mediate tissue remodeling by modifying the constitution of collagen fibers, while CCN2/CTGF induces collagen synthesis (Fig. 6) and binds to gelatin *in vitro *[21]. As such, periostin itself may collaborate with CCN2/CTGF molecule in the remodeling of periodontal ligament.

As well as at the mRNA expression level, rCCN2/CTGF also stimulated collagen synthesis (Fig. 6). The turnover of collagen in the periodontal ligament is believed to be controlled by the balance between collagen synthesis and degradation. In this respect, up-regulation of collagen synthesis by rCCN2/CTGF may be accompanied by the dynamic control of gene expression of matrix metalloproteinases (MMPs) or tissue inhibitors of matrix metalloprotainases (TIMPs) for the remodeling of periodontal ligament [42]. It should be noted that induction of particular MMPs by CCN2/CTGF was reported to occur in vascular endothelial cells. Therefore, CCN2/CTGF may play a central role in promoting the remodeling of periodontal ligament through direct and indirect actions.

According to a previous report [25], CCN2/CTGF expression was observed in dental laminas, invaginating epithelium, and condensing mesenchyme at the bud stage. Afterwards, strong expression was observed in the enamel knot and preameloblasts; and CCN2/CTGF molecules were also detected in stratum intermedium and underlying dental mesenchyme, as well as in the dental epithelium. Additionally, during experimental tooth movement, CCN2/CTGF was highly expressed in osteoblasts around the periodontal ligament [42]. In this study, we confirmed the *ccn2/ctgf *expression and cell biological effects of CCN2/CTGF *in vitro*, by using MPL cell cultures. Overall, our results support the *in vivo *findings mentioned above, together suggesting critical roles of CCN2/CTGF in the development and remodeling of dental tissues.

## Conclusion

In this study, expression of CCN2/CTGF in MPL cells was confirmed *in vitro*. Utilizing this system, we uncovered the potential of CCN2/CTGF to promote the growth and differentiation of MPL cells. These findings indicate the critical roles of CCN2/CTGF in periodontal tissue development and strongly suggest the utility of CCN2/CTGF in periodontal tissue regeneration, as already proven in articular cartilage.

## Methods

### Cells and Cell culture

The mouse periodontal ligament cell line (MPL) was established from periodontal ligament explants from extracted mandibular molars of a BALB/c mouse [27] and maintained as described [28]. The cells were grown in alpha-modified Eagle's medium (α-MEM; Nissui, Tokyo, Japan) supplemented with 10% heat-inactivated fetal bovine serum (FBS; JRH Biosciences, Lenexa, KS, USA), and kanamycin (100 μg/ml) at 37°C in a humidified atmosphere of 5% CO_2 _in air.

### Production of recombinant connective tissue growth factor (rCCN2/CTGF)

Human *ccn2/ctgf *cDNA containing the entire coding region was inserted into the plasmid expression vector pcDNA3.1(-) (Invitrogen, Carlsbad, CA, USA), and the vector was used to transform HeLa cells. The conditioned medium of the HeLa cells was concentrated and purified by heparin-affinity column and anti-CTGF/Hcs24 affinity-column chromatography. Purity was determined by immunoblotting and silver staining of SDS-PAGE gels, and highly purified fractions were used for experiments. The biological activity of rCCN2/CTGF was almost the same as that of the factor purified from a human chondrosarcoma cell line (HCS-2/8), as determined by their biological activity toward chondrocytes.

### Estimation of DNA synthesis

The mitogenic effect of rCCN2/CTGF was assessed by measuring [^3^H] thymidine (Amersham Bioscience Corp., Piscataway, NJ; Specific activity 74 TBq/nmol) incorporation into MPL cells [29,30]. The cells were labeled with [^3^H] thymidine (4.8 MBq/ml in DMEM) for 4 hrs, then washed with phosphate-buffered saline (PBS), and collected in 100 μl of PBS containing 0.25% trypsin (w/v) and 0.02% EDTA (w/v). They were next harvested onto glass-fiber paper filters (WALLAC, Turku, Finland) with a semi-automatic microharvester (TOMTEC, Yamaguchi, Japan), washed with PBS, and treated subsequently with 5% trichloroacetic acid (TCA). The radioactivity was determined with a Micro Beta PLUS (WALLAC).

### Estimation of cell proliferation

The effect of rCCN2/CTGF on cell proliferation was assessed by using Tetracolor-One (Seikagaku Corp., Tokyo, Japan) as instructed by the manufacturer.

### Northern blotting

Ten micrograms of total RNA was electrophoresed in a formaldehyde agarose gel and subsequently blotted onto a nylon membrane. For hybridization probes, CCN2/CTGF and CCN1/Cyr61 cDNA fragments were prepared by RT-PCR with pairs of specific primers. Primers specific for CCN2/CTGF [27] and CCN1/Cyr61 [28] were described previously. These PCR products were radiolabeled and used for hybridization as described earlier [20].

### Reverse transcription-polymerase chain reaction (RT-PCR)

By using ISOGEN (Nippon Gene, Tokyo, Japan)[31], we extracted total cellular RNA from MPL cell cultures treated with rCCN2/CTGF (100 ng/ml) or vehicle; and before RT-PCR, the RNA was treated with RNase-free DNase I. Total RNA (1 μg) was reverse-transcribed by using an RNA PCR kit (Perkin Elmer, Branchburg, NJ), and the cDNA was used as a template to amplify fibroblast or osteoblast-related marker genes and murine CCN2/CTGF and GAPDH genes by PCR using Taq GOLD polymerase (Perkin-Elmer). Primer sequences employed and PCR conditions were as follow:

murine glyceraldehyde 3-phosphate dehydrogenase (GAPDH), sense 5'-CACCATGGAGAAGGCCGGGG-3' and antisense 5'-GACGGACACATTGGGGGTAG-3'(418 bp); type I collagen, sense 5'-TCTCCACTCTTCTAGGTCCT-3' and antisense 5'-TTGGGTCATTTCCACATGC-3'(250 bp); osteopontin, sense 5'-ACACTTTCACTCCAATCGTCC-3' and antisense 5'-TGCCCTTTCCGTTGTTGTCC-3'(239 bp); osteocalcin, sense 5'-TCTGACAAACCTTCATGTCC-3' and antisense 5'-AAATAGTGATACCGTAGATGCG-3'(198 bp); periostin, sense 5'-TGGTTCCTCTCCTGCCCTTA-3' and antisense 5'-ACCATGCCGTGTTTCAGGTC-3'(556 bp); ALPase, sense 5'-GCCCTCTCCAAGACATATA-3' and antisense 5'-CCATGATCACGTCGATATCC-3'(372 bp); Osf-2/Cbfa-1, sense 5'-GAGGGCACAAGTTCTATCTGGA-3' and antisense 5'-GGTGGTCCGCGATGATGTC-3' (385 bp); murine CCN2/CTGF, sense 5'-GGTAAGGTCCGATTCCTACCAGG-3' and antisense 5'-CTAGAAAGGTGCAAACATGTAAC-3'(120 bp).

After pre-denaturation for 7 min at 94°C, the chain reaction was repeated for 25 cycles to maintain exponential conditions for amplifications (30 cycles for murine osteopontin). Each cycle consisted of denaturation at 94°C for 1 min, with annealing and polymerization at 60°C for 2 min. Ten-micro liter samples were analyzed on 2% agarose gels and stained with ethidium bromide along with a 100 bp-ladder molecular size marker.

### Alkaline phosphatase (ALPase) activity

The effect of rCCN2/CTGF on ALPase activity was assayed according to the procedure of Majeska *et.al*. [32]. The cells were homogenized in 0.5 ml of 0.9% NaCl and 0.2% Triton X-100 with a Polytron at 4°C, and centrifuged for 15 min at 12,000 × *g*. ALPase activity in the supernatant was measured by using *p*-nitro phenyl phosphate (*p*-NP) as a substrate. The supernatant was mixed with 0.5 M Tris-HCL buffer (pH9.0) containing 0.5 mM *p*-NP and 0.5 mM MgCl_2_. The sample was then incubated at 37°C for 30 min, and the reaction was stopped by the addition of a 0.25 volume of 1N NaOH. Hydrolysis of *p*-NP was monitored as the change in absorbance at 415 nm with a spectrometer (Amersham Bioscience Corp.). The protein concentration was determined by using the BCA protein assay system (Pierce, Rockford, IL). The activity was defined in units, 1 unit being the enzyme activity hydrolyzing 1 nmol of *p*-NP per 1 mg protein in 30 min.

### Collagen synthesis

The effect of rCCN2/CTGF on collagen synthesis was assayed by measuring the incorporation of [^3^H] proline (Amersham, 1.7 TBq/nmol) in collagenase-digestable proteins [33-35]. When MPL cells reached confluence, they were labeled with [^3^H] proline (37 MBq/ml) for 12 hrs and then collected into 1 ml of 50 mM Tris (pH7.2) containing 0.2% Triton X-100 and 1 mM phenylmethylsulfonyl fluoride (PMSF). Half of the solubilized homogenate was digested with purified bacterial collagenase for 4 hrs at 37°C, and the other half was incubated with the vehicle (control). The radioactivity in the suspended protein was measured with a liquid scintillation counter.

### In situ hybridization

For generation of the antisense probe, a 120-bp fragment of murine *ccn2/ctgf *cDNA corresponding to a non-coding region was subcloned into the pGEM-T plasmid (Promega, Madison, WI, USA), and riboprobes were synthesized by using a DIG RNA labelling kit (Roche, Mannheim, Germany) or ^35^S-UTP(30TBq/nmol, Amersham Biosciences Corp.) following the manufacturer's instructions. Sense probes were used as negative controls. After *in vitro *transcription, the labeling reaction mixture was treated with DNase I, and the probes were precipitated with ethanol, and then dissolved in 0.1% diethyl pyrocarbonate (DEPC)-treated double distilled water containing 0.01% dithiothreitol.

MPL cells cultured on glass slides were washed with PBS and fixed with 4% paraformaldehyde (PFA)- 0.1% sodium-cacodylate at 4°C for 2 hrs. The samples were washed with PBS, and digestion with 1 μg/ml proteinase K was then performed at room temperature for 10 min. After having been washed with PBS, the samples were treated with 2 mg/ml of glycine-PBS at room temperature for 10 min, and immersed in 0.1 M triethanolamine-HCl (pH 8.0). Thereafter acetic acid-anhydride (0.25% concentration) was added dropwise over a 5-min period, and incubation was then continued for another 15 min. The slides were rinsed with 4 × standard saline-citrate (SSC) for 10 min twice, and immersed in pre-hybridization buffer (3 × SSC and 50% formamide) at 70°C for 30 min for blocking.

Hybridization was performed with a hybridization mixture (50% formamide, 3 × SSC, 10% dextran sulfate, 1 μg/ml of tRNA, 1 μg/ml of sonicated salmon sperm DNA, and 1 μg/ml BSA) containing 0.4 μg/ml riboprobes at 55°C for 16 hrs in a humidified box. The slides were washed 3 times for 20 min each time with 2 × SSC and 50% formamide at 55°C, while being shaken at 90 strokes/min to remove the excess riboprobe; and then they were incubated at 37°C for 30 min with 20 μg/ml of RNase A in NTE buffer [0.5 mM NaCl, 10 mM Tris-HCl (pH 8.0) and 1 mM EDTA]. After having been rinsed in NTE buffer at 37°C for 5 min, the slides were washed 3 times for 20 min each time with 0.5 × SSC at 37°C and incubated at room temperature for 30 min in TBS buffer [100 mM Tris-HCl (pH 9.5), 100 mM NaCl and 150 mM NaCl] containing 1% normal goat serum. Next, the sections were incubated with 1/500-diluted alkaline phosphatase (AP)-conjugated sheep anti-digoxigenin antibody at room temperature for 1 hr. To visualize digoxigenin-labeled riboprobe-tissue mRNA complexes, we incubated the sections in a freshly prepared solution of nitro blue tetrazolium chloride (NBT) and 5-bromo-4-chloro-3-indolylphosphate (BCIP) in an alkaline buffer [100 mM Tris-HCl (pH9.5), 100 mM NaCl and 50 mM MgCl_2_] containing 1 mM levamisole in a dark room at room temperature. Finally, the slides were washed in distilled water 3 times and mounted in aqueous mounting material.

### Statistical methods

The statistical difference between treated or control samples was assessed with Student's *t*-test. All experiments were repeated at least twice, and similar results were obtained.

## Competing interests

The author(s) declare that they have no competing interests.

## Authors' contributions

MA performed molecular biological studies and prepared most of the data. SK rearranged and drafted the manuscript. T Nakanishi arranged the constitution of the work. T Nishida designed a significant portion of the experiments. TY and TS established and participated in the *in situ *hybridization analysis. GY and KO collaborated in performing most of the cell biological analyses. YM provided general support. MT conceived and designed the study and drafted the manuscript.
